# Contextualizing Changes in e-Cigarette Use During the Early COVID-19 Pandemic and Accompanying Infodemic (“So Much Contradictory Evidence”): Qualitative Document Analysis of Reddit Forums

**DOI:** 10.2196/66010

**Published:** 2025-03-20

**Authors:** Shannon Lea Watkins, Katherine Snodgrass, Lexi Fahrion, Emily Shaw

**Affiliations:** 1 Department of Community and Behavioral Health College of Public Health University of Iowa Iowa City, IA United States; 2 College of Public Health University of Iowa Iowa City, IA United States

**Keywords:** vaping, nicotine, tobacco, health communication, social media, new media

## Abstract

**Background:**

Understanding how social media platforms facilitate information exchange and influence behavior during health crises can enhance public health responses during times of uncertainty. While some risk factors for COVID-19 susceptibility and severity (eg, old age) were clear, whether e-cigarette use increased risk was not clear. People who used e-cigarettes had to navigate both the COVID-19 infodemic and a conflicting, politicized, and changing information environment about the interaction between COVID-19 and e-cigarette use.

**Objective:**

This study aims to characterize and contextualize e-cigarette–related behavior changes during the early COVID-19 pandemic and illuminate the role that social media played in decision-making.

**Methods:**

We conducted a qualitative analysis of COVID-19–related e-cigarette discussions on 3 Reddit forums about e-cigarettes. We collected 189 relevant discussion threads made in the first 6 months of the pandemic (collected from June 27, 2020, to July 3, 2020). Threads included 3155 total comments (mean 17 comments) from approximately 1200 unique Redditors. We developed and applied emergent codes related to e-cigarette perceptions and behaviors (eg, the role of nicotine in COVID-19 and do-it-yourself narratives) and web-based community interactions (eg, advice), identified thematic patterns across codes, and developed a model to synthesize the socioecological context of e-cigarette behaviors.

**Results:**

e-Cigarette subreddits provided a platform for Redditors to discuss perceptions and experiences with e-cigarettes, make sense of information, and provide emotional support. Discussions reflected an array of e-cigarette–related behavioral responses, including increases and decreases in use intensity, changes in purchasing practices (eg, stockpiling), and changes in vaping practices (eg, reusing disposable pods). This study presented a theoretically and empirically informed model of how circumstances created by the pandemic (eg, changes in activity space and product shortages) compelled behavior changes. Redditors drew from their existing perceptions, intentions, and experiences with nicotine and tobacco products; their personal pandemic experiences; and their participation on Reddit to decide whether and how to change their e-cigarette behaviors during the early pandemic. Forums reflected uncertainty, stress, and debate about the rapidly evolving and complicated public health information. Consumption and discussion of media (eg, news articles and peer-reviewed publications) on Reddit informed e-cigarette perceptions and behaviors. Decisions were complicated by distrust of the media.

**Conclusions:**

Variations in individual traits and environmental circumstances during the early COVID-19 pandemic provide context for why there was no unified direction of e-cigarette behavior change during this period. Information and discussion on Reddit also informed risk perceptions and decisions during the pandemic. Social media is an effective and important place to communicate public health information, particularly during crisis or disaster situations. Moving forward, transparent, accurate, and specific message development should consider the stress, struggles, and stigma of people who use e-cigarettes and address the roles mistrust and misinformation play in decisions.

## Introduction

### Background

During the first year of the COVID-19 pandemic, risk perceptions, related behaviors, and public health guidance changed rapidly, as both the virus and our understanding of it evolved. The COVID-19 pandemic was accompanied by an “infodemic,” a glut of accurate and inaccurate information about a disease outbreak, which can cause confusion and undermine public trust [1-3]. Conflicts and changes in public health messaging confused some members of the public and seemed untrustworthy to others [4,5].

In this era of high stress, evolving public health information, and politicization of science, the public used social media as a tool to inform their health-related decisions [6]. During crises and disasters, social media facilitates information dissemination and communication but can also facilitate the intentional and unintentional spread of information that is “inaccurate, incomplete, vague, or ambiguous,” [7] that is, *misinformation* [8-10]. During the intense isolation of the early pandemic, social media also provided essential human connection. In 2020, nearly one-third of Americans reported viewing COVID-19–related content on social media daily, and almost two-thirds of social media users reported they were unlikely to fact-check information about COVID-19 they read on social media with a health professional [6]. Understanding how social media platforms facilitate information exchange and impact behavior during health crises is critical to improving infodemic management and future public health response [1]. Moreover, social media conversations provide a unique record of the human experience during the pandemic.

Globally, the pandemic influenced multiple addictive behaviors, including alcohol consumption [11,12], gambling [13], cannabis use [14,15], and tobacco and nicotine use [12,16-20]. Evidence suggests that nicotine e-cigarette use, purchasing, and risk perception were all impacted [21] and that changes were mixed (ie, toward e-cigarette use and away from it) for both adolescents and adults [22-25]. e-Cigarette use is influenced by personal beliefs and experiences and interpersonal, informational, organizational, community, and policy contexts (ie, socioecological context) [26]. Influences include risk perceptions, social norms [27], e-cigarette advertising [28], social media [29], and access to e-cigarettes [30]. By transforming multiple domains of an individual’s life (eg, activity space and social connections), the pandemic likely influenced e-cigarette use through multiple pathways [31].

People who used e-cigarettes exchanged e-cigarette–related information on the web during the pandemic [4,32], leveraging already-established social media forums on which individuals discussed products, flavors, current regulations, and personal experiences [33]. While some risk factors for COVID-19 susceptibility and severity, such as old age, were clear, whether e-cigarette and tobacco use increased risk was not clear [34-36]. People who used e-cigarettes or tobacco products not only had to navigate the COVID-19 infodemic but also make sense of conflicting, politicized, and changing information about the interaction between COVID-19 and e-cigarette and other nicotine and tobacco product use [37]. The mixed effect of the pandemic on e-cigarette use, the compounding and interacting uncertainties about COVID-19 and e-cigarette use, and the existing e-cigarette communities on the web suggest e-cigarette use is an excellent context to investigate how social media captured lived experience during the pandemic and how it facilitates health-related information exchange during public health crises. The COVID-19 pandemic will influence health and well-being long after the threat of severe illness subsides. Documenting *how* the profound personal and social changes of the pandemic influenced health risk behaviors in the early months of the crisis can reveal facilitators and barriers to health promotion, help us contextualize and interpret behavioral trends as they unfold in the years following, and suggest future communication interventions during crises.

### Objectives

We aimed to explicate the mechanisms of e-cigarette behavior change, including engagement in web-based forums, during the early months of the COVID-19 pandemic. We leveraged social media as a source of qualitative data and conceptualized social media use as a phenomenon of study, conducting a qualitative analysis of e-cigarette forums on the social media platform Reddit (Reddit, Inc). Understanding the discourse in web-based e-cigarette communities during the COVID-19 pandemic can illuminate the range of individual experiences of vaping during that time; show how health information, including misinformation, is consumed, analyzed, and implemented on the web; and illuminate how individuals make health decisions during crisis.

## Methods

We thematically analyzed randomly selected Reddit posts (posted from January 31, 2020, to July 3, 2020) related to the COVID-19 pandemic from 3 e-cigarette subreddits.

### Study Site

Reddit is a social media site that allows anonymous users (Redditors) to post content on discussion boards (subreddits) dedicated to specific topics. Redditors interact by creating an original post (OP) related to the subreddit, commenting on an OP, and “upvoting” (liking) or “downvoting” (disliking) OPs and comments. The term OP refers to both *original posts* and the Redditor who created that OP. Because each subreddit is dedicated to a specific topic, it provides space for relevant discussions, questions, and advice. Each month, approximately 500 million visitors participate on Reddit, and the site hosts 73 million unique users daily [38]. Because of these attributes, Reddit has been effectively used to investigate e-cigarette perceptions and behaviors [39-42] and is uniquely situated to study web-based social interactions during the pandemic through unobtrusive observation. A large study of psychosocial language markers in health discussions on Reddit reported substantial differences in topic and sentiment before and during the pandemic [43]. This qualitative study included 3 e-cigarette subreddits over 6 months to capture a range of reactions to the pandemic.

### Data Collection

**Figure 1:**
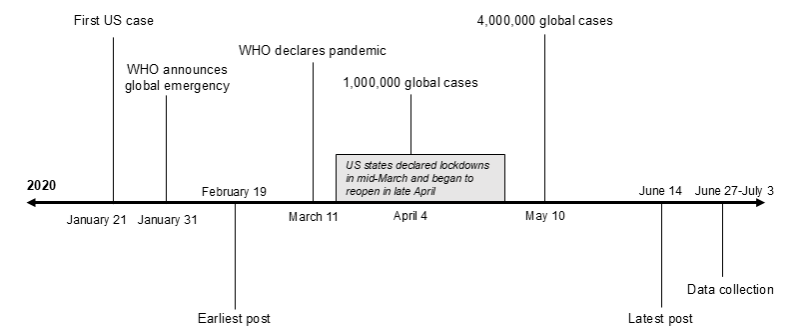
A timeline of events related to the COVID-19 pandemic in early 2020. We collected Reddit data from June 27, 2020, to July 3, 2020. The final dataset included original posts made between February 19, 2020, and June 14, 2020. WHO: World Health Organization.

In each subreddit, we searched for OPs using 8 terms: “COVID,” “corona,” “coronavirus,” “pandemic,” “quarantine,” “virus,” “lockdown,” and “isolation.” For inclusion, OPs had to be written in English and posted no earlier than January 31, 2020, when the United States declared the novel coronavirus a public health emergency. KS conducted searches from June 27, 2020, to July 3, 2020. We captured a range of reactions to COVID-19, as our data collection window included many other important dates, including the virus being named COVID-19 on February 11, Italy’s lockdown beginning on February 23, the first community spread case in the United States on February 26, the first report of a US death on February 29, and the declaration of a pandemic on March 11. Figure 1 presents a timeline of COVID-19 pandemic events, data collection, and date range of OPs. Posts had to relate to both e-cigarette use and the COVID-19 pandemic. Because posts were made in e-cigarette subreddits, mentions of “vaping” were assumed to be related to nicotine vaping unless there was an explicit mention of cannabis vaping and no mention of nicotine vaping. To capture interactions between Redditors, eligible posts had to have at least 1 comment from another Redditor. We excluded OPs that mentioned cannabis vaping and made no reference to nicotine vaping or e-cigarettes.

For each search, we screened OPs randomly (to decrease algorithmic bias where newer or more popular posts appeared first) until we collected 10 relevant posts and all comments (ie, threads) or reviewed all search results. KS saved 189 conversation threads (PDF) and recorded search metadata, including search terms, number of hits, number of ineligible posts removed before hitting 10 relevant posts, search date, and qualitative observations about the search (eg, June 27 entry for search term *coronavirus,* “I’m noticing individuals like to leave articles with little to no commentary and then ask for others’ interpretations. In the comments, a robust discussion often occurs. It is interesting to see individuals using others’ interpretations/community discussion.”). When saved, OPs had an average of 17 comments (range 1-167; total: n=3155), an average of 25 “points” (assigned by Reddit), and were 81% upvoted on average. Approximately 1200 unique usernames engaged in conversation. If a Redditor deletes their account, their comment remains but their username does not. This occurred infrequently in our dataset. Still, we could not determine how many additional Redditors participated.

### Data Analysis

We conducted a qualitative thematic analysis to (1) contextualize e-cigarette–related behavior changes during the early COVID-19 pandemic and (2) characterize web-based discussions about e-cigarettes during that time. After the first complete reading of the data, KS and ES led codebook development using an iterative process of reading, note-taking, and discussion to identify recurrent ideas and come to an agreement on codes and definitions. They developed an initial codebook of inductive codes, definitions, and examples; tested the codebook; and refined it to ensure comprehensiveness and clarity between codes. SLW provided suggestions and mentorship throughout the process. The final codebook included 7 codes related to e-cigarette perceptions and behaviors and 3 codes related to web-based community interactions. Finally, we used a 3-tiered quality code of “priority,” “average,” and “low priority” to indicate richness (a qualitative determination based on the number, length, and detail of posts) of the thread’s content.

KS completed coding and led the analysis. We applied codes to threads in their entirety to preserve the interrelated, unique interactions between Redditors within each threaded conversation, and codes were assigned based on relevant content in the OP, comments, or both. We assigned multiple codes to a single thread when relevant and used Microsoft Excel to track coding.

For each code, KS combined all relevant threads into a single document (PDF, then converted to a Microsoft Word document) and performed data reduction, a process that involved reading each post in its entirety, summarizing relevant content, saving illustrative quotes, and keeping analytic notes to document emerging ideas and interpretations. For each code, we used summaries, illustrative quotes, and analytic notes to identify patterns. *Priority* threads were analyzed first, and *average* threads were read if posts continued to add unique information to our analysis (ie, saturation was not yet met). KS and SLW used the collaborative web-based whiteboarding application Miro to organize cases and analytic ideas, returning to original texts to test emerging themes. During this analytic process, we used the socioecological model—which posits that individual behavior is influenced by their social, physical, and cultural environment—as an organizing framework to tease apart the external and internal factors Redditors mentioned. This iterative process produced the final model and thematic findings presented subsequently.

### Ethical Considerations

This study was determined not to be human participants research by the University of Iowa Institutional Review Board (project title “202006140 e-cigarette conversations in online social media platforms”). All posts in our dataset were accessible on the web and might be considered publicly available data. Furthermore, Redditors select usernames, which can conceal their identity. However, they did not formally consent to the inclusion of their comments in research and might not have conceptualized their posts as truly “public.” To avoid the reidentification of contributors, we used only short quotes in our examples, did not name the subreddits we collected data from, and did not identify Redditors by their usernames. We did not engage with Redditors for this study. Documents were stored on shared cloud storage through the University of Iowa and accessible only to team members.

## Results

### Overview

**Figure 2:**
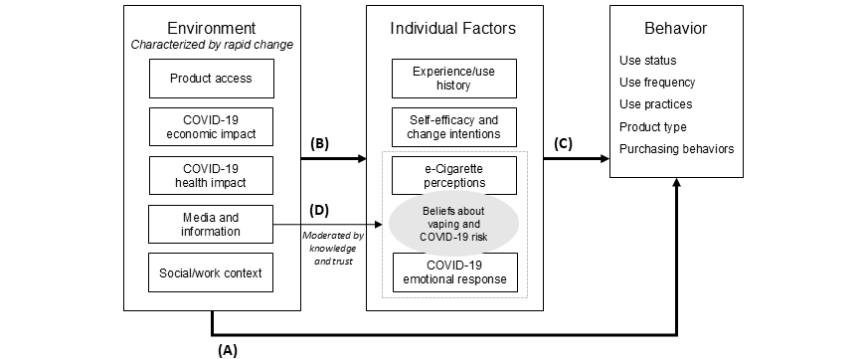
A model of environmental and individual influences on 5 categories of behavior, developed through qualitative document analysis, visualizes the complexity of the pandemic’s downstream impact. Changes in circumstances during the early COVID-19 pandemic compelled behavior changes (A) and changed perceptions and intentions (B), which in some instances also led to behavior change (C). Information and misinformation in the media and social media environment (D), especially information about the risks of COVID-19 for people who use e-cigarettes and tobacco, indirectly influenced behavior change through changes in perceptions of risk. Participation on Reddit itself exposed Redditors to information and advice of varying quality, which in turn might have impacted product perceptions and use practices. Findings are based on a qualitative analysis of 189 discussion threads from Reddit, posted between February 19, 2020, and June 14, 2020, and collected from June 27, 2020, to July 3, 2020.

Reddit served as an information hub, debate platform, public journal, and a place of emotional support for people who used e-cigarettes early in the COVID-19 pandemic. Our analysis reflected the multifaceted experiences of people who use e-cigarettes, represented in a model of the environmental and individual influences on behavior change discussed by Redditors (Figure 2). Our findings indicate that circumstances created by the pandemic compelled behavior changes as they affected the context, pace, and environment in which individuals experienced life. Redditors drew from their existing perceptions, intentions, and experiences with e-cigarettes and cigarettes; their pandemic experience; and their participation on Reddit to decide whether and how to change their e-cigarette behaviors. Consumption of media and information (eg, peer-reviewed publications) and associated commentary shared on Reddit played a role in shaping behavior. Posts revealed how decisions were complicated by media distrust and by tobacco regulatory context.

### Reddit as a Source of Support, Information, Debate, and Influence

Rapidly evolving public health information about COVID-19 created uncertainty, stress, and confusion. The subreddits acted as community spaces for individuals seeking to express these emotions and receive advice and support. Subreddit posts during the pandemic included solicitation and sharing of e-cigarette knowledge. For example, one Redditor who could not afford to replace their broken device sought expertise from other Redditors, stating “All advice is appreciated.” Suggestions included power cycling (ie, turning off and turning on again), ensuring the coil of the device was not damaged, and checking the charging pins. Other OPs asked subjective questions, such as advice on whether they should quit vaping because of the pandemic. One Redditor who reported having asthma and being a “pretty heavy vaper” explained they feared a more severe illness but a lack of “solid evidence” about vaping made them unsure about what they should do, so they asked others for their opinions.

Reddit also served as a space for discussion of information from other media, such as news articles, research publications, and blog posts related to the COVID-19 pandemic. The discourse about media seemed to change opinions for some Redditors, while for others it confirmed their existing beliefs. For example, when a commenter questioned a study’s validity because it was published in the 1940s, the OP acknowledged the observation was a “good call.” Another Redditor described propylene glycol (PG) and vegetable glycerin in detail, inaccurately claiming that vegetable glycerin had antimicrobial and antiviral properties, to which another Redditor expressed gratitude for the information.

Redditors shared anecdotes and stories, and other Redditors used these stories to form ideas about COVID-19. In response to an OP wondering whether they should quit vaping during the pandemic, one Redditor opined that there is “no evidence” of a relationship between COVID-19 and vaping because their whole family contracted COVID-19 except for them, and they were the only one in their family who vaped. The anecdote was used to support the OP’s decision to continue vaping.

Comments providing emotional support or sharing experiences in solidarity reflected a sense of community. For example, one OP experiencing nicotine withdrawal asked whether their symptoms were normal, and a commenter affirmed their experience and assured them they would “wake up more and more happy” over time. Another Redditor received 6 recommendations after requesting e-cigarette device options for their mother who had lost her husband to COVID-19 and “desperately want[ed] to quit [cigarettes].” Posts reflected solidarity with people who were using e-cigarettes as a harm reduction strategy, encouraging people to avoid relapse (eg, “stay strong and don’t do it”) or advocating for vape shops to remain open to support the people who “rely on vape shops to make sure they don’t return to cigarettes” and the many people who are “one cigarette away from giving up on vaping.” Many Redditors disagreed, arguing their closure was important for COVID-19 prevention.

### Behavior Changes and Their Contexts

#### Overview

Thematic analysis revealed how the early COVID-19 pandemic directly and indirectly motivated changes in e-cigarette use and e-cigarette practices. We found no consistent pattern of change; some Redditors reported increasing use, and others reported decreasing or pausing use (ie, at least temporary cessation). Synthesizing our thematic analysis, Figure 2 visualizes pandemic-related behavior changes as influenced by environmental factors (ie, physical, economic, and legal environment that constrained individual behavior) and personal characteristics (ie, individual perceptions and experiences with e-cigarettes), which could be modified during the pandemic by personal experiences and exposure to information and misinformation, including Reddit participation. Posts indicated that the impact of environmental context, consumed media, and personal experience depended on prepandemic risk perceptions and behaviors.

#### Direct Impacts of the COVID-19 Pandemic on e-Cigarette Behaviors

Redditors explicitly and implicitly referred to life changes during the COVID-19 pandemic, including working from home, job loss and financial insecurity, exposure to COVID-19, and reduced access to e-cigarettes. These changes in circumstances led to changes in behavior. For example, one Redditor reported having fewer hours of work and therefore more time to play video games. Their already-established practice of vaping while playing video games meant they increased their e-cigarette use when their gameplay increased. A Redditor who previously smoked cigarettes and lived in a state with a flavored product ban lost access to their usual flavored e-cigarette products when they stopped commuting to work across state lines. Because of this loss of access, they were considering reinitiating cigarette smoking. One Redditor who used e-cigarettes socially and could not spend time with friends because of the COVID-19 pandemic social distancing restrictions reported they “stop[ped] vaping for a while,” although they implied that they might start again. In contrast, a few Redditors who worked in essential services or already worked from home cited their lack of job change as the reason they had not changed their vaping intensity.

COVID-19 itself prompted behavior change, largely temporary reduction or cessation, for a few Redditors who reported COVID-19 symptoms, such as shortness of breath, a cough, or fatigue that made vaping difficult or impossible. One Redditor with a “terrible cough” switched to nicotine patches, and another decreased use when COVID-19–like symptoms made vapor inhalation difficult. Some posts revealed potentially harmful practices, like one Redditor who diluted e-liquid with water and consumed it orally while they had a cough and suggested that other people who were sick try the same.

Several posts reflected e-cigarette changes in response to other effects of COVID-19, such as the loss of a loved one or high test positivity rates, including one account of increased vaping while coping with the loss of their grandfather to COVID-19.

The economic impact of the early pandemic left some Redditors with less discretionary income to spend on e-cigarettes. For example, one Redditor shared that they had not worked in a week, ran out of e-liquid, and had < US $1 left in their bank account. Because they could no longer afford e-cigarette products, they “resorted” to their fiancé’s cigarettes, sharing their disgust with the emojis “😖🤢.”

**Figure 3:**
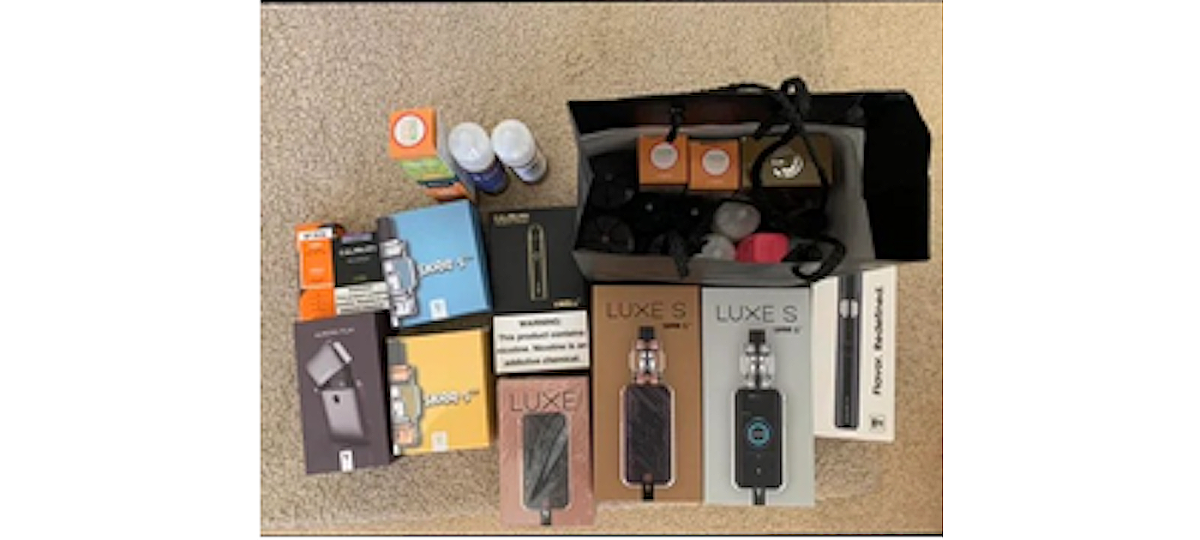
e-Cigarette stockpile shared on Reddit.

Purchasing patterns adapted to changing circumstances; vape shop closures, curbside pickups, web-based purchases, limited supplies, and shipping delays altered purchasing behaviors. Some individuals began “stockpiling” e-cigarette devices and accessories because of real or anticipated shortages (Figure 3). In response to observed or anticipated reduction in access, some Redditors altered their use and personal practices, including reducing volume (eg, abstinence when unable to buy their favorite e-liquid) and changing products (eg, turning to pipe tobacco after running out of pods and e-liquid because they were “still addicted”). Redditors reported “DIY” activities in the face of shortages, for example mixing their own e-liquid from a bulk purchase.

Redditors referenced potentially risky adaptations, such as using other wires in place of standard product coils, attempting their own device repairs, or trying to clean disposable pods to reuse. Replies to these posts included encouragement, suggestions, and discouragement. One Redditor who realized they bought expired e-liquid still considered vaping it because it was their only option. Another shared that their local shop was selling 3-year expired juice with fake stickers to look fresh; some responses suggested it might be usable, but most responses strongly discouraged this behavior and some suggested reporting the retailer.

A Redditor shared an image of their stockpile after going to their local vape shop. The owner of the store was in the process of closing for the pandemic, but the Redditor shared they felt prepared (because of their stockpile), asserting “Bring on the lockdown.”

Reddit discussions reflected an array of e-cigarette–related behavioral responses to the COVID-19 pandemic and infodemic, including increases and decreases in use intensity, changes in purchasing practices (eg, stockpiling), and changes in vaping practices (eg, reusing disposable pods). These findings are based on a qualitative analysis of 189 discussion threads from Reddit, posted between February 19, 2020, and June 14, 2020, and collected from June 27, 2020, to July 3, 2020.

People who used e-cigarettes were impacted by both the pandemic response and the existing e-cigarette regulatory scheme in their locale. A few Redditors made references to changing e-cigarette policy or stay-at-home orders, illustrating this impact. For example, some individuals had already stockpiled devices, e-liquid, or accessories in response to regulatory changes, and thus were not as severely impacted by the pandemic.

#### Impacts of Individual Perceptions and e-Cigarette or Cigarette History on e-Cigarette Behaviors

Two key beliefs anchored risk perception during the pandemic: (1) COVID-19 was a serious illness and (2) e-cigarette use increased COVID-19–related risk. The belief that e-cigarette use made COVID-19 more dangerous (either in disease susceptibility or severity) sufficiently increased the perceived risk of vaping to prompt behavior change for some Redditors. For example, one Redditor stopped vaping entirely because of an underlying belief that vaping was not “exactly 100% safe and healthy” and a desire for their body not to “suffer unnecessarily” if they contracted COVID-19. Others reduced their e-cigarette use “just in case” using e-cigarettes increased COVID-19 risk or severity. One Redditor who had COVID-19 shared that they felt their vaping did not make their symptoms worse but still took a 2-week hiatus when their symptoms were severe. Alternatively, Redditors who did not perceive both COVID-19 and e-cigarettes as risky were unlikely to alter their behavior. Some Redditors perceived COVID-19 as a nonserious or nonexistent condition. Others believed they had negligible exposure and were therefore unconcerned about contracting COVID-19. This lack of concern meant no change in behavior for one Redditor who asserted they would continue to vape alone at home. While washing their hands. And not touching their face (punctuation in the original comment).

Perceived addiction, previous cigarette smoking, existing intentions to quit, and self-efficacy to change behavior help explain the extent to which people who used e-cigarettes changed their behavior in response to the pandemic. One Redditor’s post reflected a tension between underlying intentions to quit and low self-efficacy, describing the pandemic as “the perfect reason” to fulfill their quit intentions but admitting that it has been hard due to the stress they had experienced. Another Redditor who read a report that vaping increased the severity of COVID-19 decided that it would be “way too difficult…[to quit] during a stressful time,” even after the study made them believe quitting might be beneficial. Product shortages left one Redditor who had previously smoked with what he saw as 2 unappealing options: “go cold turkey” or “return to cigarettes.”

### e-Cigarette– and COVID-19–Related Beliefs and Their Development

The complex environment of COVID-19 and e-cigarette information during this time resulted in an array of interpretations of the health risks that COVID-19 posed to people who used e-cigarettes. Redditors offered multiple explanations for why e-cigarette use disproportionately increased the health risks for people who used e-cigarettes, including COVID-19 and vaping impacting the same bodily systems, the vape device itself acting as a transmission vector, or nicotine harmfully impacting the angiotensin converting enzyme 2 (ACE2; a protein) receptor system. For example, one Redditor expressed concern with vaping during the pandemic, stating nicotine may increase the number of ACE2 receptors, which are the “[receptors] this specific virus hijacks to get into cells.” However, some Redditors suggested vaping may be beneficial for fighting or preventing COVID-19. One Redditor described how studies show inhaling PG can “prevent you from getting the flu” and argued PG has “incredible antimicrobial effects,” reporting they would continue vaping. Another said “nicotine [and PG] kill bacteria,” concluding that vaping was beneficial. Others made claims without proposing a mechanism, such as “vaping...help[s] against pneumonia.”

Some posts reflected the salience of former smoking on e-cigarette– and COVID-19–related beliefs. For example, in response to an OP who asked whether concern about the risks of vaping and COVID-19 was causing people to stop vaping, several Redditors commented on how vaping “makes a huge difference” (compared to smoking cigarettes) on the severity or frequency of respiratory infections, implying that the relative wellbeing is evidence that vaping does not increase COVID-19 risks.

Reddit threads served as a forum to discuss information about the COVID-19 pandemic, and Redditors cited information from a variety of sources, including public health authorities and scientific organizations, traditional media sources, such as newspapers and televised broadcasts, and informal news sources, such as blog and social media posts. The thread mentioned earlier, which included the OP asking about the risk of vaping (submitted on March 18, 2020) and 120 comments, offers an interesting case of how Redditors pieced together the range of questions about vaping-related risks. The tone and quality of the debate varied across comments, which included personal anecdotes, scientific claims, and commentary on the quality of the discussion. Many posts included unsubstantiated claims. Other comments supported claims with a range of sources, including articles from PubMed, a publicly accessible database of scientific and medical research funded by the US federal government. Some comments asked for evidence to support others’ claims, offered corrections, and identified logical fallacies (eg, noting the claim that vaping was better for respiratory health than smoking did not address the question of whether, in the absence of smoking, vaping was better than not). One post asked the subreddit to avoid circulating opinions and stay very close to high-quality sources of information to protect public safety, and a comment on another thread noted to the OP that the subreddit was the not the best place to ask about perceptions about vaping and covid risk, “seeing as everyone is going to have a biased opinion.”

As we have documented, Redditors credited beliefs about whether and how COVID-19 and e-cigarette risk interacted for driving their behavior change. In some instances, it was apparent that the conversations in subreddits influenced the development of these beliefs. Some conversations suggested that the media individuals consumed had a profound impact on their perceptions, thoughts, and behaviors during this time. Perhaps most colorfully, one Redditor commented, “HOLY FUCK. There is so much contradictory evidence. I’m like a sheep. I read one comment and put my [vape] away. I read another and pick it up! I just need to stop using this shit.”

We found evidence that individual factors moderated one’s interpretation and understanding of Reddit and media content (Figure 2). Perceived contradictions between sources, a Redditor’s level of trust in each source, assumptions, and understanding of the scientific process altered how Redditors weighed new information. For some Redditors unfamiliar with the research process, distrust emerged when recommendations for pandemic safety changed as additional research was published.

The underlying trust of an information source influenced a Redditor’s assessment of risk regarding COVID-19, vaping, and their intersection. When an OP asked whether they should vape less during the pandemic, a commenter said no, stating “all the bs” in the media is grounded in “zero actual science.” Another Redditor complained that officials “refer to all nicotine intake as ‘smoking’” and emerging studies regarding ACE2 receptors and smoking show “how incompetent and ignorant” officials are in the United States. These generalizations across nicotine delivery methods and seemingly conflicting scientific evidence likely deepened the distrust of the media for this individual.

## Discussion

### Principal Findings

Our qualitative thematic analysis of e-cigarette–related conversations on Reddit during the early months of the COVID-19 pandemic revealed changes in amount, frequency, product type, purchasing behaviors, and use practices from the widespread effects of the COVID-19 pandemic. By examining the “nuanced deliberations” [40] in social media communities of people who use e-cigarettes, our study illuminates how the variety in behavioral responses during the pandemic evolved in response to socioecological changes, individual nicotine and tobacco product use history, perceptions and beliefs, the information environment, and the interactions between these factors.

Our finding of mixed behavioral changes from the pandemic’s impact on everyday life is consistent with other reports about e-cigarettes [24,44], and we hope our model can help piece together the growing work on the contextual and individual factors [45] to explain *why* behavior change was so varied. On the one hand, Reddit conversations suggested that reduced access to e-cigarette products, salient health messages and concerns [46,47] (although Klemperer et al [48] report mixed findings), and changes in circumstance encouraged cutting back and quitting. On the other hand, stress, boredom, unstable routines, and changes in activity space (eg, working from home [49]) encouraged more nicotine use [50] or deterred cessation. Other findings suggest that for individuals trying to quit, pandemic-related stress might have undermined coping skills and therefore suppressed cessation [51]. Studies on alcohol consumption [52] and cigarette smoking [12] suggest variations in the types and sources of stress may explain some of the divergence in e-cigarette use behavior. Our observation that some Redditors were concerned about product access and stockpiled vaping materials early in the pandemic is consistent with previous reports about e-cigarettes [53] and resembled stockpiling of essential goods (eg, meat and toilet paper). The similarity suggests that these individuals considered e-cigarettes an essential good.

Our research builds upon previous work that identifies social media as a key space for individuals to seek health-related advice, information, and community, demonstrating in the context of vaping during the pandemic how the community-building and knowledge-sharing functions of social media can affect people’s health behavior. In another Reddit study, Sharma et al [40] found that people with mental illness perceived that web-based discussion about the shared interest in vaping facilitated social connectedness for them. We speculate that during the early months of the COVID-19 pandemic, when other socializing may have been scarce and access to substances was limited, Reddit and other social media platforms were particularly important for connectedness and support among individuals who use substances [54-56]. Still, using social media has also been associated with cigarette and e-cigarette use [29,57,58], and an intervention during the first year of the pandemic reported that a 30-minute reduction in daily social media use decreased smoking behavior [59].

Misinformation can easily circulate on the web because the credibility of information on social media cannot be certified, and social media algorithms create “echo chambers.” The Online Misinformation Engagement Framework identifies four stages in which people engage with web-based misinformation as follows: (1) selecting information sources, (2) choosing to consume or ignore the information they are exposed to, (3) evaluating that information (ie, evaluation), and (4) engaging with the information (ie, reaction). In times of scientific and social uncertainty, when public health messaging is limited and competing with misinformation, people might turn to social media communities seeking advice, information, and strategies from people they perceive as trustworthy (ie, the pandemic might have impacted stage 1: source selection) [1]. Our findings suggest Reddit served as a trusted source of information about e-cigarettes for community members during the early pandemic. Previous work has documented social media conversations about the intersection of vaping and COVID-19, including health concerns and unsubstantiated claims (Twitter [60] and YouTube [32]). In the discussion we observed, some Redditors critically evaluated the claims (stage 3: evaluation) and engaged in peer-to-peer debunking (stage 4: reaction); however, we do not know the extent to which all viewers engaged in these behaviors and whether these actions impacted perceptions of those reading.

There remains no definitive information about the intersection of COVID-19 and vaping risk [36]. Some claims in our dataset do appear to be supported by scientific evidence [36]. In addition, it is likely that Redditors were sincere in their discussion, as evidence suggests that people rarely share misinformation intentionally. Still, most posts engaged with just one or a few dimensions of this complicated question, and it appears individuals made decisions based on incomplete information. Cognitive bias might also be at play. A UK study reported that adults who used e-cigarettes were more likely to assert that using e-cigarettes decreased or did not affect the risk of severe COVID-19 symptoms [34], although empirical evidence of the effects was uncertain. The conversations we analyzed likely reflected and potentially perpetuated this optimism bias. In addition, misinformation (including incomplete information) might spread more easily in vaping subreddits, which engage a relatively homogenous network than a more general subreddit with more diverse participants [61].

We posit that Reddit conversations impacted behavior both directly (eg, providing advice for vape repair) and indirectly by informing beliefs (including both accurate and inaccurate perceptions). Consistent with our finding that information on Reddit influenced decision-making for some Redditors, a study of college students found that exposure to and seeking COVID-19–related news was related to lower odds of pausing smoking or vaping [62]. We speculate that the sometimes heated discussion of information, including misinformation, in these subreddits might have increased some Redditors’ already heightened pandemic-related stress and perhaps contributed to vaping escalation or avoided reduction in vaping intensity. Moreover, exposure to inaccurate information might have a lasting impact; for example, Redditors who vaped more believing that it would decrease their odds of contracting COVID-19 would inadvertently increase their e-cigarette–related health risks. An observational study of people who had tried to quit using e-cigarettes reported higher relapse among people who recalled at least one misinformation claim about COVID-19 and nicotine [63]. Other information might have also increased the risk. Redditors who followed suggestions on how to extend the life of their e-cigarette devices (eg, making their own coil) might have been exposed to more toxic chemicals. Studies on the health effects of improper use of e-cigarette devices can inform our understanding of the extent to which the pandemic not only influenced use frequency but changed the risk profile of e-cigarette use.

Our study demonstrates the value of qualitative work in behavioral research, as it illuminates the nuance of how often, how much, or in what way people use vaping products. Our qualitative analysis elucidates the array of socioecological and personal considerations Redditors had at the time. This array of experiences might help explain conflicting findings between quantitative studies with different samples [17,64]. It also suggests that, because summary statistics obscure bidirectional impacts, cross-sectional analyses will likely underestimate the extent to which e-cigarette practices were impacted during the crisis. Longitudinal, quantitative surveillance will determine how widespread and long-lasting the pandemic’s impacts were on vaping at a population level. Qualitative work can contextualize those impacts. In addition, while quantitizing social media data can reflect overall discussion topics [43], qualitative analysis can reveal the community dynamics, such as the role of personal anecdotes on risk perceptions, that play out in those discussions.

The lack of unified messaging about e-cigarettes reflected on Reddit is not a new problem and follows a decade of conflicting messages. The e-cigarette industry has advertised e-cigarettes as a cigarette cessation device. Scientific evidence is mixed on their effectiveness for cessation and relative health benefits. The outbreak of e-cigarette, or vaping, product use–associated lung injury resulted in a growing but unsubstantiated perception that e-cigarettes may be more dangerous than combustible cigarettes [65]. There is a need for consistent, easy-to-understand messaging to fight misinformation on the web about COVID-19 and the risks and practices associated with e-cigarettes. Applying the tools of infodemic management might improve vaping-related communication [3]. Although an experimental study reported that US adults perceived messages that linked smoking to COVID-19 as effective for decreasing vaping [66], our findings suggest that messages and evidence specific to e-cigarette use rather than tobacco products overall would be viewed as more trustworthy and acceptable. Leveraging many communication channels can be an effective way to reach target audiences with antivaping campaigns [67], and Reddit itself might be a potential avenue for health communication interventions [68].

Moreover, e-cigarette interventions might address underlying causes of substance use, misinformation, and distrust. Much of our data revealed the impact of stress, coping, mental health struggles, and feelings of stigmatization, including from public health officials. We expect cessation messaging that acknowledges the experiences and struggles of individuals who use e-cigarettes and the meaning that e-cigarettes have in their lives (eg, stress relief) might better resonate with this population. This and other work highlight the role of community dynamics of social media in information exchange; health communication might leverage these relationships to disseminate accurate health information. Structural- and individual-level misinformation interventions across stages of engagement (eg, content labels, introducing friction that encourages the viewer to pause before sharing information) will also likely improve the quality of decision-making [69] about vaping.

### Limitations

Our analysis aimed to document e-cigarette behavior change and reveal mechanisms through which the early pandemic and related infodemic impacted behavior; our findings may be an incomplete list or overemphasize the experiences of more active Redditors. Moreover, many individuals may not have changed their e-cigarette use behavior at all and the behavior changes that we report may have been only temporary.

The unobtrusive methods that allowed us to observe Reddit conversations were limited in several ways. Because we did not communicate with Redditors, our ability to contextualize narratives in the specific national and local policy and social norms was restricted to the information Redditors disclosed, we are unable to determine how long changes lasted, and we cannot describe our sample’s demographic characteristics or baseline use behaviors. Redditors are predominantly White and male; therefore, it is likely that our study overemphasized their experiences [70,71]. Pandemic-related stressors might have intersected with and compounded experiences of minority groups’ stress [72,73] and resulted in increased vaping [74-76]; by contrast, resilience, optimism, and sense of community might have buffered some individuals with minoritized identities from that stress [77,78]. Work that investigates the digital experiences of minoritized groups during the pandemic is essential [79]. Our team is composed of female researchers in the United States; therefore, we might have missed cultural cues outside of our experience or expertise. We have not documented how perceptions and behaviors have evolved since June 2020 as new information emerged, policies changed, and more recent waves of the pandemic occurred. Although the specific perceptions about the relationship between vaping and COVID-19 might not be transferable today, we believe our findings speak to broader questions about the role of social media in information exchange during a crisis and the relationship between people who use e-cigarettes and public health.

### Conclusions

This study elucidated how people make decisions about their health during a time of informational and social uncertainty. Information is gathered from a variety of sources, increasingly from social media platforms like Reddit, making public health research on these platforms critical. We were able to gain insight into the way people processed and discussed information and identify individual and environmental changes (eg, local laws and supply chain) that provide an explanation for why there was no unified direction of e-cigarette behavior change during the early pandemic. Social media is an effective and important place to communicate public health information, particularly during crisis or disaster situations, and it is crucial that e-cigarette information is accurate, transparent, accessible, nonstigmatizing, and specific to the target population.

## Abbreviations

ACE2: angiotensin converting enzyme 2
OP: original post
PG: propylene glycol

## Data Availability

The dataset generated and analyzed during this study is not publicly available; although Reddit posts are publicly available and anonymized by username, Redditors did not consent for their content to be shared publicly in a curated dataset. The dataset is available from the corresponding author on reasonable request for research purposes.
